# Supplementary material for: SPO11 dimers are sufficient to catalyse DNA double-strand breaks in vitro
Source: Nature. 2025 Feb 19;639(8055):792–9. doi: 10.1038/s41586-024-08574-8 (PMC11922746; doi:10.1038/s41586-024-08574-8)
Supplement: Supplementary file 1 — Supplementary discussion, tables and data. [file 41586_2024_8574_MOESM1_ESM.pdf]

nature portfolio

https://doi.org/10.1038/s41586-024-08574-8

Supplementary information

SPO11 dimers are sufficient to catalyse DNA double-strand breaks in vitro

In the format provided by the authors and unedited

Supplementary Discussion

1. A model for the formation of meiotic DNA double strand breaks

In all eukaryotes where this has been investigated, SPO11 requires a cohort of accessory partners to catalyze break formation *in vivo*[3,4,41]. *In vitro*, SPO11 cleaves DNA independently, but only at high protein/DNA ratios, which we interpret as facilitating encounters between SPO11 monomers on the DNA substrate. *In vivo*, high DNA concentrations will effectively titrate SPO11 monomers, preventing dimerization and cleavage. Thus, we propose that a primary role of the accessory partners is to control the timing and localization of SPO11 dimerization.

In mice, the known essential partners of SPO11 include TOP6BL, REC114, MEI4, IHO1 and MEI1 (RMMI) (**Extended Data Fig. 9a**)[12-16]. We have shown that SPO11-TOP6BL forms a 1:1 complex with cleavage activity comparable to that of SPO11 alone, although this is dependent on experimental conditions. In addition, we previously showed that the yeast orthologs of REC114, MEI4, IHO1 (Rec114, Mei4 and Mer2) assemble DNA-dependent condensates *in vitro* that recruit the yeast Spo11 core complex (Spo11, Ski8, Rec102, Rec104)[37]. Despite extreme sequence divergence, the structure of these proteins is widely conserved across eukaryotes, although their DNA-binding and condensation activities appear to vary quantitatively between organisms[42,43]. Hence, we propose that the mouse RMMI proteins control SPO11 cleavage by assembling biomolecular condensates within which SPO11-TOP6BL is recruited (**Extended Data Fig. 9b**). Recruitment of SPO11-TOP6BL complexes likely involves the interaction between a TOP6BL C-terminal α-helix that binds the pleckstrin homology (PH) fold of REC114[31]. Partitioning of SPO11-TOP6BL complexes within these condensates would allow them to reach a critical threshold necessary for dimerization and DSB formation.

In both yeast and mice, SPO11 frequently catalyzes multiple closely-spaced DSBs, in particular in the absence of the DNA-damage response kinase Tel1/ATM[39,40,44], consistent with the local accumulation of SPO11 within condensates[37]. The distance between these coincident breaks ranges from about 30 nt to several kb, and the more closely-spaced events show a 10-bp periodicity, indicative of co-orientation of SPO11 dimers on the DNA substrate[39,40]. Thus, condensates likely enhance the local concentration of SPO11 and facilitate the co-orientation of SPO11 proteins, promoting productive dimerization on the DNA substrate (**Extended Data Fig. 9c**).

2. Target site selection

In mice, the DSB landscape is mostly defined by sequence-specific binding of PRDM9[17-19]. Within DSB hotspots, the selection of cleavage sites is likely influenced by SPO11 itself, but whether this is indeed the case has not been demonstrated. In yeast, mutations within the DNA-binding grove of Spo11 affect the fine-scale DSB landscape, indicating that Spo11 directly influences target site selection[10,29].

Our experiments show that SPO11 cleavage is affected by the sequence and topology of DNA *in vitro*. The effect of DNA sequence on the cleavage activity may arise from base-discriminating contacts between the protein and the substrate and/or through indirect effects of the DNA sequence on structural features of the double helix, such as bendability. The impact of DNA topology could stem from DNA intertwining (which is unlikely, as a SPO11 dimer presumably senses only one duplex at a time), underwinding, or bending.

Hence, three scenarios could explain our observations: (i) SPO11 is influenced by direct base-discriminating contacts and a preference for underwound DNA, (ii) only DNA bendability matters, or (iii) SPO11 cleavage is affected by a combination of these factors. Evidence supporting the latter scenario includes AFM images showing yeast and mouse SPO11 complexes bound to a bent DNA substrate[29,35]. Additionally, the yeast Spo11 complex also binds with higher affinity to DNA mini-circles that induce tension on the double helix[29], and a cryo-EM structure of the yeast complex bound to a gapped substrate shows a 130° angle between the duplexes[34]. Furthermore, AlphaFold models of SPO11-TOP6BL complexes from

mice and other organisms show SPO11 dimers that accommodate underwound and bent DNA (**Fig 5b**, **Extended Data Fig. 10**). In addition, Topo VI has also been shown to actively bend DNA *in vitro*[33] and DNA bending by topoisomerase II activates DNA cleavage[45]. Finally, base discriminating contacts have been observed in a cryo-EM structure of the yeast Spo11 complex[34], with the implicated residue (K173) conserved in mice (K175), also found to contact DNA bases in AlphaFold models[35]. Collectively, these data suggest that base-discriminating contacts, underwinding of DNA strands, and bending of the substrate all likely impact DNA cleavage by SPO11.

The preference of SPO11 for bendable and negatively supercoiled DNA could be an evolutionary remnant of the substrate preference of Topo VI, imposed by structural constraints of the complex. Alternatively, this property may have been conserved for its functional consequences, potentially linking DSB formation to topological stress. Another possibility is that the requirement for DNA bending prior to cleavage constitutes an activation barrier, with stabilization of the post-cleavage complex into a lower-energy state aiding the progression of the reaction (**Extended Data Fig. 9d**, further discussed below).

3. DNA nicking

In addition to the expected double-strand cleavage activity, we show that mouse SPO11 frequently makes single-stranded DNA breaks *in vitro*, which is evident in reactions that do not result in complete plasmid consumption. This indicates that the cleavage of the two DNA strands is not strictly coordinated. Within a SPO11 dimer, cleavage of one DNA strand could occur independently from the other; alternatively, the first cleavage could facilitate or somewhat hinder the second. Our current data does not allow us to discriminate between these scenarios.

The accumulation of nicked products may result from the weak dimer interface of SPO11. In this scenario, a fraction of SPO11 dimers that have catalyze the first strand cleavage may collapse before the second strand is cleaved.

Whether nicks also accumulate during meiosis has not been definitively established. However, previous attempts to detect single-stranded DNA breaks at meiotic hotspots in *S. cerevisiae* failed to reveal such products[7,46,47]. Perhaps the presence of accessory partners during meiosis stabilizes SPO11 complexes, preventing a premature collapse before catalysis is completed.

4. Plasmid relaxation

Our experiments also reveal that SPO11 has plasmid relaxation activity *in vitro*. This activity was detected in conditions that accumulate single-stranded DNA breaks, for example with mixtures of active and inactive proteins. The DNA relaxation activity most likely occurs through a swiveling mechanism caused either by the dissociation of the SPO11 dimer interface or the dissociation of one monomer from DNA, which would result in a rotation the DNA double helix around the intact phosphodiester bond. Restauration of the dimeric complex would then allow the reversal of the cleavage reaction and the liberation of a partially relaxed covalently closed plasmid.

This plasmid relaxation activity was not anticipated based on the biological functions of SPO11. Indeed, it is weak and only observed in specific conditions, suggesting it may have limited physiological relevance. Nevertheless, it demonstrates that the cleavage reaction is inherently reversible, consistent with the transesterase mechanism of SPO11, since the energy of the broken phosphodiester bond is maintained in the covalent complex.

5. Reversal of DNA cleavage

While there is currently no direct evidence for DNA religation *in vivo*, it has long been proposed that the formation of DSBs would be preceded by a reversible state[1,26]. Possible evidence for this comes from ChIP-qPCR analyses of *S. cerevisiae* Spo11 that identified a formaldehyde-independent hotspot DNA signal in a *rad50S* mutant that preceded the accumulation of DSBs measured by Southern blot analysis[48]. This signal was sensitive to salt concentration in the extraction buffer and required the catalytic tyrosine of Spo11, leading the authors to suggest

reversible cleavage as an attractive explanation. A possible interpretation for their findings is that the transition of cleavage products from a reversible to an irreversible state is delayed in a *rad50S* mutant, and that the extraction protocol in this ChIP assay indeed captured covalent complexes in their reversible state. Cleavage reversal could be accelerated at high salt concentrations, allowing the DNA to be released before precipitation. Similarly, when the covalent complex of Topo II is trapped in the presence of Ca^{2+}, increasing the salt concentration stimulates the religation activity[49].

This scenario implies that direct visualization of DSBs by Southern blot analysis may fail to capture early covalent complexes due to the rapid reversal of the reaction during DNA extraction.

6. The transition to an irreversible break

What then triggers the transition from a reversible to an irreversible break? We propose that this occurs through the relaxation of SPO11 on the broken DNA ends, which would cause, or be triggered by, the collapse of the dimer interface. Indeed, cryo-EM analysis of the yeast Spo11 complex bound to a DNA end indicates that the configuration of Spo11 in this 'post-cleavage' complex is incompatible with DNA cleavage[34]. The transition from a reversible covalent complex to an irreversible post-cleavage complex would entail a 45° rotation of the Toprim domain with respect to the WH domain, which would not be compatible with dimerization. This transition would separate the two ends of the DSB, a necessary step for detection by MRN/X complex and activation of the endonuclease activity[50].

Thermodynamically, the pre-cleavage and reversible covalent complexes correspond to high-energy states (**Extended Data Fig. 9d**). The activation barrier required to assemble a pre-cleavage complex may in part be imposed by the necessity to bend the DNA duplex and could be overcome by the energy provided by topological stress. Following cleavage, the transition to a lower-energy state would therefore drive the irreversibility of the reaction. Consistent with this interpretation, both yeast and mouse complexes exhibit higher affinity for DNA ends compared to duplex substrates, suggesting that the end-bound complex represents a lower-energy state[29,35]. In contrast, mouse SPO11 protein alone does not bind efficiently to DNA ends, indicating that TOP6BL plays a crucial role in this transition.

The strong end-binding activity of SPO11-TOP6BL further suggests that the complex could remain bound to the DNA ends after resection has initiated, potentially impacting DSB repair. Indeed, mapping ssDNA-dsDNA junctions in mouse meiosis previously identified signatures compatible with a SPO11-bound recombination intermediate[51,52].

Supplementary References

41 Lam, I. & Keeney, S. Mechanism and regulation of meiotic recombination initiation. *Cold Spring Harb Perspect Biol* **7**, a016634, doi:10.1101/cshperspect.a016634 (2015).

42 Daccache, D. *et al.* Evolutionary conservation of the structure and function of meiotic Rec114-Mei4 and Mer2 complexes. *Genes Dev* **37**, 535-553, doi:10.1101/gad.350462.123 (2023).

43 Liu, K. *et al.* Structure and DNA-bridging activity of the essential Rec114-Mei4 trimer interface. *Genes Dev* **37**, 518-534, doi:10.1101/gad.350461.123 (2023).

44 Lukaszewicz, A., Lange, J., Keeney, S. & Jasin, M. De novo deletions and duplications at recombination hotspots in mouse germlines. *Cell* **184**, 5970-5984.e5918, doi:10.1016/j.cell.2021.10.025 (2021).

45 Lee, S. *et al.* DNA cleavage and opening reactions of human topoisomerase IIα are regulated via Mg2+-mediated dynamic bending of gate-DNA. *Proc Natl Acad Sci U S A* **109**, 2925-2930, doi:10.1073/pnas.1115704109 (2012).

46 de Massy, B., Rocco, V. & Nicolas, A. The nucleotide mapping of DNA double-strand breaks at the CYS3 initiation site of meiotic recombination in Saccharomyces cerevisiae. *Embo j* **14**, 4589-4598 (1995).

47 Xu, L. & Kleckner, N. Sequence non-specific double-strand breaks and interhomolog interactions prior to double-strand break formation at a meiotic recombination hot spot in yeast. *Embo J* **14**, 5115-5128 (1995).
48 Prieler, S., Penkner, A., Borde, V. & Klein, F. The control of Spo11's interaction with meiotic recombination hotspots. *Genes Dev* **19**, 255-269, doi:10.1101/gad.321105 (2005).
49 Osheroff, N. & Zechiedrich, E. L. Calcium-promoted DNA cleavage by eukaryotic topoisomerase II: trapping the covalent enzyme-DNA complex in an active form. *Biochemistry* **26**, 4303-4309, doi:10.1021/bi00388a018 (1987).
50 Reginato, G. *et al.* HLTF disrupts Cas9-DNA post-cleavage complexes to allow DNA break processing. *Nat Commun* **15**, 5789, doi:10.1038/s41467-024-50080-y (2024).
51 Paiano, J. *et al.* ATM and PRDM9 regulate SPO11-bound recombination intermediates during meiosis. *Nat Commun* **11**, 857, doi:10.1038/s41467-020-14654-w (2020).
52 Yamada, S. *et al.* Molecular structures and mechanisms of DNA break processing in mouse meiosis. *Genes Dev* **34**, 806-818, doi:10.1101/gad.336032.119 (2020).

Supplementary Tables

Plasmid name	Description
pCCB628	HisFlag-TOP6BL in pFastBac1
pCCB630	MBP-SPO11 in pFastBac1
pCCB642	MBP-SPO11(Y137F,Y138F) in pFastBac1
pCCB1084	MBP-SPO11(Y137F) in pFastBac1
pCCB1085	MBP-SPO11(Y138F) in pFastBac1
pCCB1086	MBP-SPO11(E224A) in pFastBac1
pCCB959	Standard 3-kb substrate for cleavage assay
pOC157	Substrate with 24× Widom 601 sequences (pBS_24x-601_MA1+MA2, Addgene plasmid #114361)
pUC19	Control substrate with 0× Widom 601
pCCB1106	pUC19 with 1× Widom 601
pCCB1107	pUC19 with 3× Widom 601
pCCB1108	pUC19 with 6× Widom 601

Table S1: Plasmids used in this study.

Oligo	Sequence
cb095	CTAGTATAGAGCCGGCGCGCCATGTCTAGATAGCGTTAGGTCTGCCGAATAGTACTA CTCGGATCCCGAGCGAACCACGC
cb100	GCGTGGTTCGCTCGGGATCCGAGTAGTACTATTCGGCAGACCTAACGCTATCTAGAC ATGGCGCGCCGGCTCTATACTAG
cb886	CTACTAAGCGTGACATCTTCTTCACCGACTCCCAGCTGTTC
cb887	GAACAGCTGGGAGTCGGTGAAGAAGATGTCACGCTTAGTAG
cb957	TAGCAATGTAATCGTCTATGACGTTAACGTCATAGACGATTACATTGC
cb1577	TACACCGACTCCCAGCTG
cb1578	GAAGATGTCACGCTTAGTAG
cb1579	TTCACCGACTCCCAGCTG
cb1580	GTAGATGTCACGCTTAGTAG
cb1581	CGAAGGACGCTACCTTC
cb1582	CCACGATCAGCAGGAAC
cb1593	CTAGTATAGAGCCGGCGCGCCATGTCTAGATAGCGTTAGGTCTGCCGAATAGTACTA CTCGGATCCCGAGCGAACCACGC-6FAM
dd77	6FAM- CTAGTATAGAGCCGGCGCGCCATGTCTAGATAGCGTTAGGTCTGCCGAATAGTACTA CTCGGATCCCGAGCGAACCACGC
pl068	GCTAATCCTGTTACCAGTGG
vg001	6FAM-GGGATTTTGGTCATGAG

Table S2: Oligonucleotides used in this study.

gBlock	Sequence
SPO11	GGATCCGAATTCACCATGGCTTTCGCCCCTATGGGTCCCGAGGCCTCCTTCTTC<br>GACGCTCTGGACAGGCACAGAGCCAGCCTGCTGGCTATGGTGAAGCGCGGAG<br>CCGGTGAAACCCCTGCTGGAGCTACTAGGGTGGCTTCCAGCTCTGAGGTCCTG<br>ACCGCTATCGAAAACATCATCCAGGACATCATCAAGTCTCTGGCCAGGAACGAG<br>GTGCCTGCTTTCACTATCGACAACAGATCATCCTGGGAAAACATCATGTTCGAC<br>GACTCTGTCGGACTGAGGATGATCCCTCAGTGCACCACTCGCAAGATCCGTTCA<br>GACTCCCCCAAGTCAGTGAAGAAGTTCGCCCTGATCCTGAAGGTCCTGTCTATG<br>ATCTACAAGCTGATCCAGTCAGACACCTACGCTACTAAGCGTGACATCTACTACA<br>CCGACTCCCAGCTGTTCGGTAACCAGGCTGCCGTGGACTCCGCCATCGACGAC<br>ATCAGCTGCATGCTGAAGGTGCCTAGGCGTAGCCTGCACGTCCTGAGCACTTCT<br>AAGGGTCTGATCGCTGGCAACCTGAGGTACATGGAGGAAGACGGCACCAGAGT<br>GCAGTGCACTTGCAGCGCTACCGCCACTGCTGTCCCAACCAACATCCAGGGAA<br>TGCAGCACCTGATCACTGACGCCAAGTTCCTGCTGATCGTGGAGAAGGACGCTA<br>CCTTCCAGAGGCTGCTGGACGACAACTTCTGCTCTAGAATGTCACCCTGCATCA<br>TGGTGACCGGCAAGGGAGTCCCAGACCTGAACACTCGCCTGCTGGTCAAGAAG<br>CTGTGGGACACCTTCCACATCCCTGTGTTCACTCTGGTGGACGCCGACCCCTAC<br>GGAATCGAGATCATGTGCATCTACAAGTACGGTTCAATGTCCATGAGCTTCGAA<br>GCTCACAACCTGACCATCCCAACTATCCGCTGGCTGGGCCTGCTGCCTTCTGAC<br>ATCCAGCGTCTGAACATCCCAAAGGACTCACTGATCCCTCTGACCAAGCACGAC<br>CAGATGAAGCTGGACTCCATCCTGAAGCGCCCCTACATCACTTACCAGCCACTG<br>TGGAAGAAGGAGCTGGAAATGATGGCTGACTCCAAGATGAAGGCCGAAATCCA<br>GGCTCTGACCCTGCTGAGCTCTGACTACCTGAGCCGCGTGTACCTGCCTAACAA<br>GCTGCGTTTCGGTGGCTGGATCTAAGTCGAC
TOP6BL	GGATCCGAATTCACCATGGAAAGGACCGCCCTGGCTGTGTGCGAGATCCTGAG<br>ATACCTGATCATCCACTGGAAGTGCGAAGCCGGCACCGCTAAGGGAACTCTGCT<br>GGACGGCCAGCTGGTCATCAGCATCGAGGCTCTGAGGTCTAAGCACCTGCCAG<br>ACTCACTGCACTGCATCATCACCATCGCCAGCACTAGATCTGTCTACGGTGGCC<br>TGAACTTCAAGAAGTTCCTGCAGGAAATCCAGCCTGCTCTCCCAAGGCTGTCTG<br>CTAAGCTGGCTCTGGCTAGCGAGGAAGGAGGTCGTAGCCAGGACGCCTCTGGA<br>ATCGCTCCTTGCCAGGTGACCTTCGAGGTGGACGAAAACTCCCAGAGCCTGAT<br>GACTGACTGCCTGGTCATCAAGCACTTCCTGCGCAAGATCATCATCGTCCACCA<br>CAAGCTGAAGTTCTCTTTCTCAGTGGCTGTCAACGGCACCCTGTCTGCCGAGAC<br>TTTCGGAGCTGAGAACGAACCTACCCTGAGGCTGGACAACGGAGTGACTCTGG<br>TGGTCGGTTTCCAGAGATACGTCAGCAAGCCCAAGCTGAACTGGAGCGAAGCT<br>CACTGCTCTCGTATCCACCCAGTGCTGGGACACCCAGCCCCTCTGTTCATCCCT<br>GACGCCAAGGCTGACACCGGTCTGCTGGGTGAACTGACCCTGACCCCTGCTGC<br>TGCTCTGTGCCCATCCCCAAAGGGTTTCTCCAGCCAGCTGTGCAGGATCTCTTC<br>AGTCAGCATCTTCCTGTACGGACCACTGGGTCTGCCTCTGCTGTCCAGCGACCA<br>GGACCAGCCTTCTACCGCTGTGTTCAGAGACACTTCATACTTCATCGACTGGAA<br>GAAGTACAACCTGTTCATGGTCCCCAACCTGGACCTGAACCTGGACACCCAGTC<br>CGTGCTGCCTGACGTCAACTACAAGGCTGAGTCCCCCGAAGGAAACCAGAGCC<br>AGAACATGAACGCCCAGGGTCCCGCTCTGCTGCTGTTCCTGTTCGTGGACTTCC<br>AGTCTGACGTGCCAGTCCAGCAGGCCAAGATCTGGGGTCTGCACACCCTGCTG<br>ACTGCCCACCTGTCAGCTATCCTGTCCGAAAGCCGCTCTACCGTCCAGCAGTCA<br>ATCCAGTCCGCTGTGGACCAGGTCTGGCAGCTGTACCACCACGACGCCAAGAC<br>TCAGCAGCGTCTGCAGGCCTCACTGTCCGTGGCTGTCAACAGCATCATGTCTGT<br>GCTGACCGGCTCCACTAGGTCTTCATTCAGAAAGACCTGCCTGCAGGCTCTGGA<br>GGCCGCTGACACTCAGGAGTTCGGAGTCAAGCTGCACAGGATCTTCTACGACAT<br>CACCCAGCACCAGTTCCTGAAGCACTGCTCTTGCGACACCGAGCAGCACCTGA<br>CTCCAGAAAAGAACATCTCAGCCCAGAACACTAAGGACCAGCACAAGAACATCG<br>CTCAGGAGTTCCCTGAGGAATCCATCGGTCAGGCCGAGAACAAGCGCCCCAAG<br>CGTGGTAGCCCAAACCACGGCCGCGAGGAATCACGTGTGCTCGGCTCCGCTCG<br>CGACCGTTCACCTCCCAAGTCCGCTACCAGGGACAGAGAGCTGACCGAAGTCT<br>CTCTGACTGCTCGCGGTTCACAGACTCAGGCTGCTCACGGAAGGGCTCAAGCT<br>GCTGAGGCTGCTTCTCCTGCTGGCGGACTGGAGGACCTGTGGCTGCAGGAAGT<br>GTCAAACCTGTCCGAGTGGCTGAACCCAGGCCACCGCAGCTAAGTCGAC

Table S3: Synthetic genes used in this study.

Supplementary Data

Provided as separate files:
AlphaFold3 models of DNA-bound SPO11 complexes from *M. musculus*, *H. sapiens*, *Z. mays*, *A. thaliana*, *D. rerio*, *D. melanogaster*, *C. elegans*, *S. cerevisiae*, *S. pombe*, and *S. macrospora*.

Supplementary Figure 1: Source data.

Uncropped gels for
- Fig. 1: pages 8-9.
- Fig. 2: pages 10-11.
- Fig. 3: pages 12-13.
- Fig. 4: pages 14-15.
- Fig. 5: page 16.
- Extended Data Fig. 1: page 17.
- Extended Data Fig. 2: page 18.
- Extended Data Fig. 3: page 19.
- Extended Data Fig. 4: page 20.
- Extended Data Fig. 5: pages 21-22.
- Extended Data Fig. 6: pages 23-24.
- Extended Data Fig. 7: pages 25-26.

Supplementary Figure 1

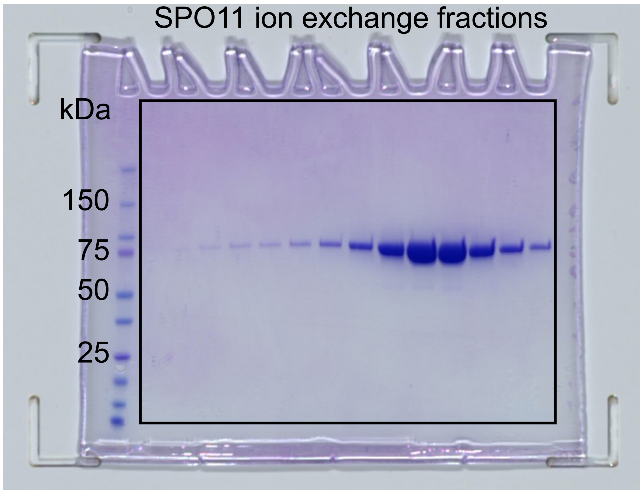

Used in Fig. 1b

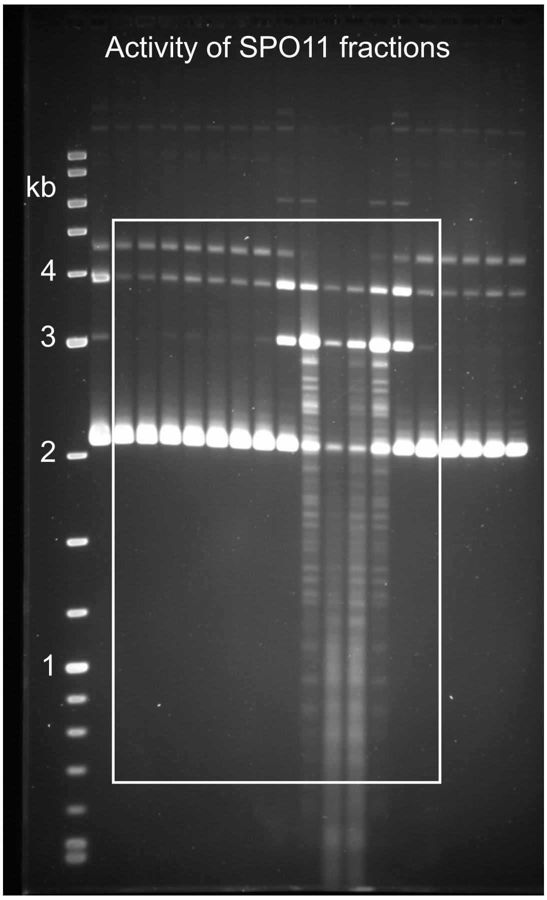

Used in Fig. 1d

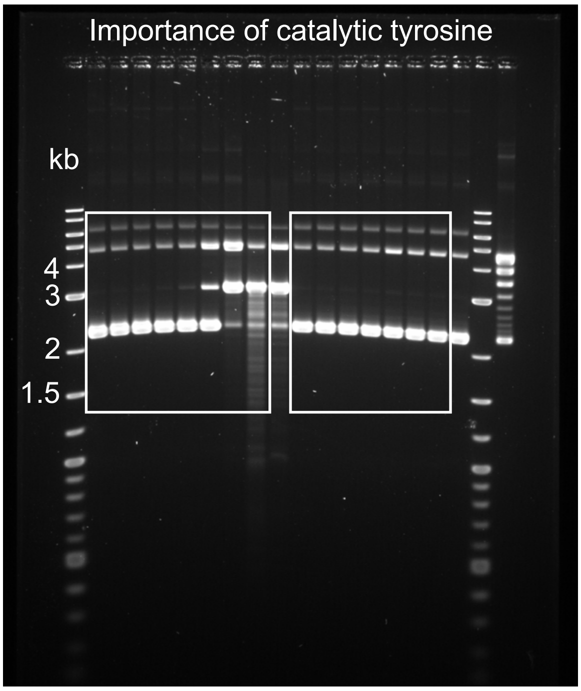

Used in Fig. 1e

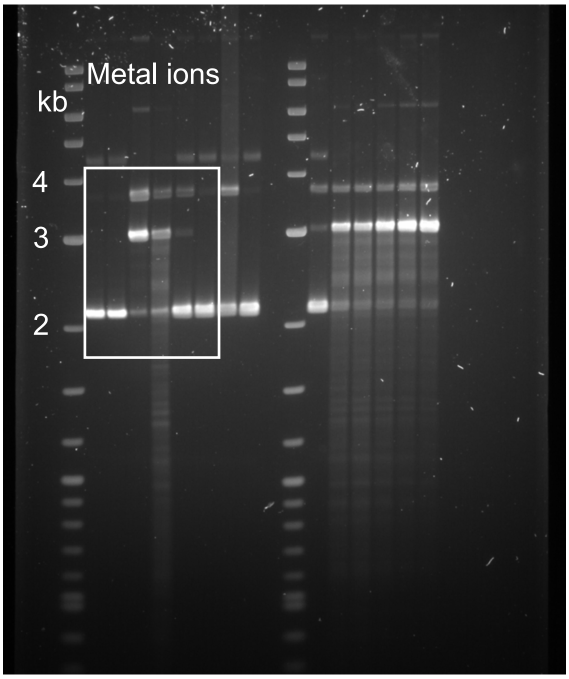

Used in Fig. 1f

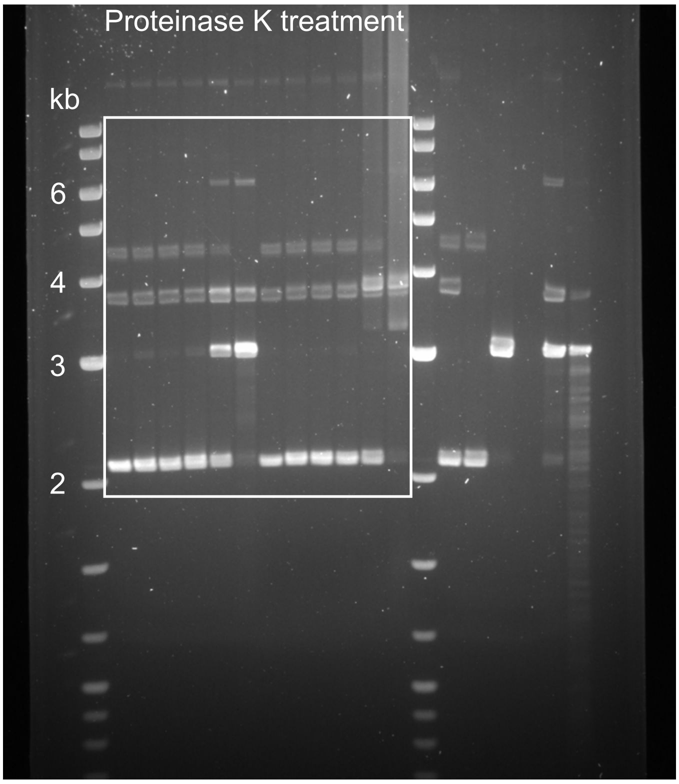

Used in Fig. 2b

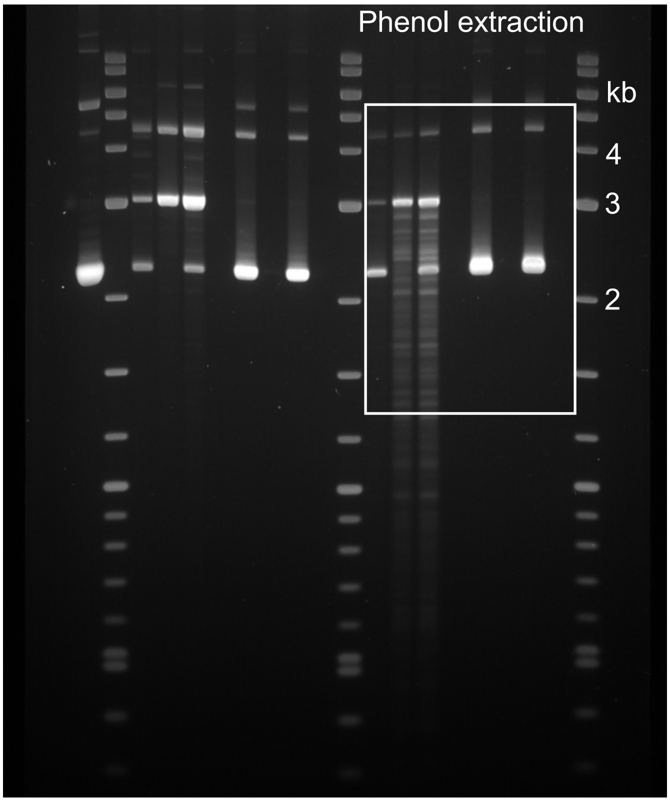

Used in Fig. 2c

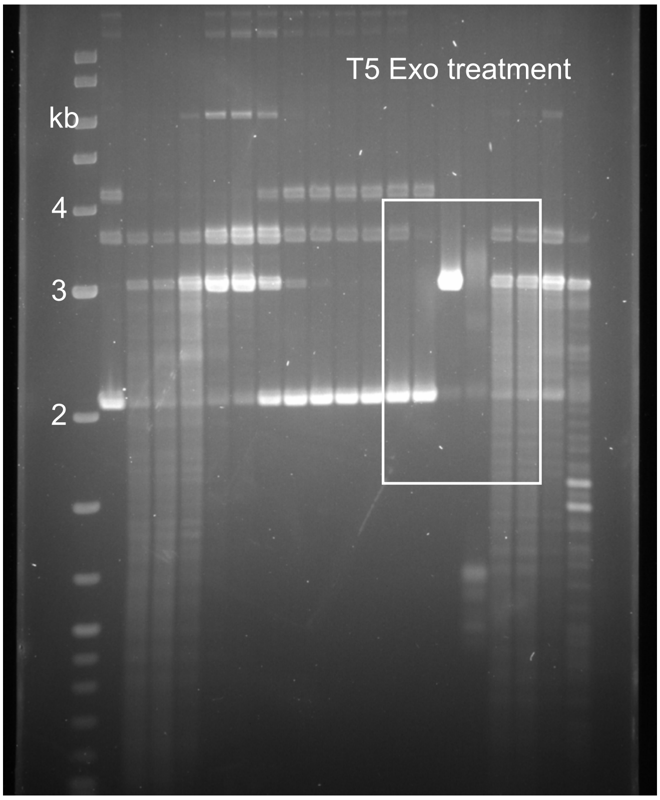

Used in Fig. 2d

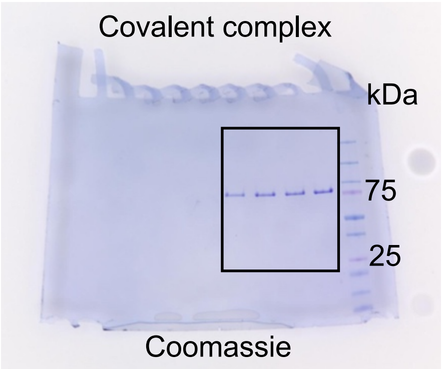

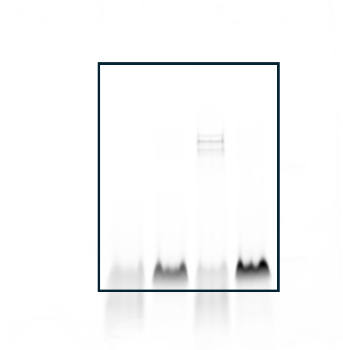

Fluorescence

Used in Fig. 2e

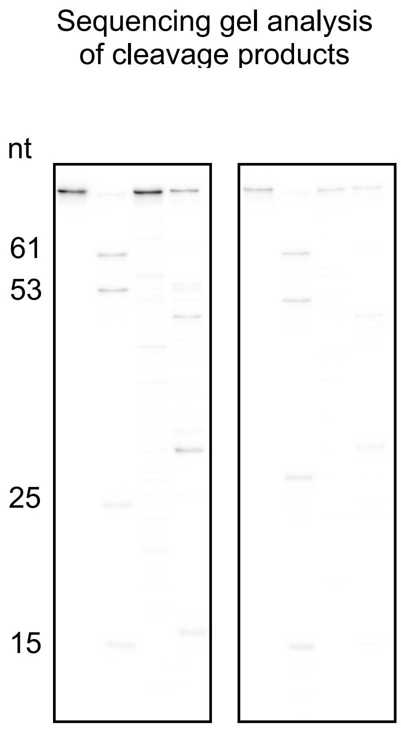

Used in Fig. 3a

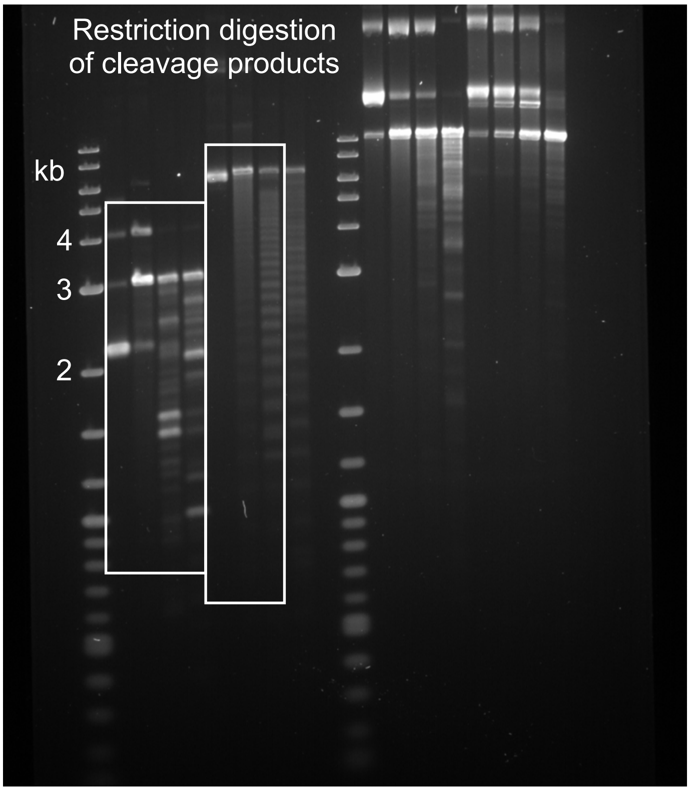

Used in Figs. 3b, c

Impact of DNA supercoiling

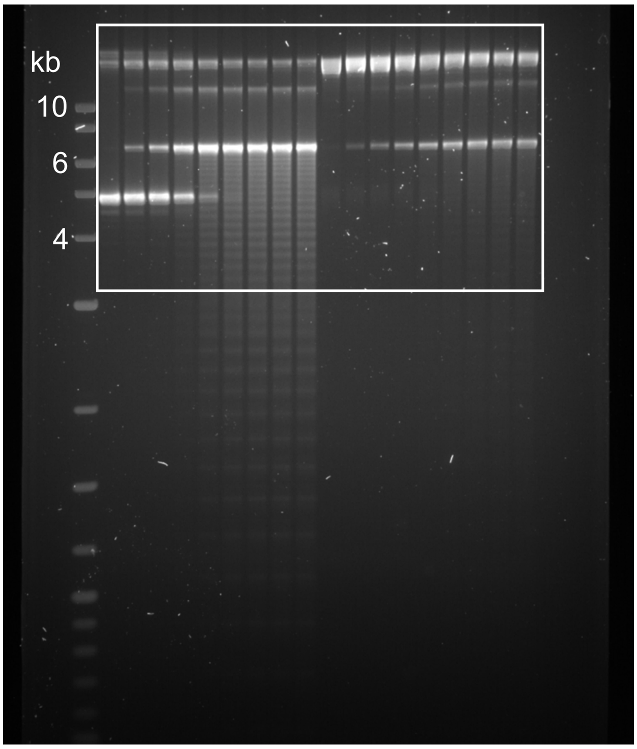

Used in Fig. 3d

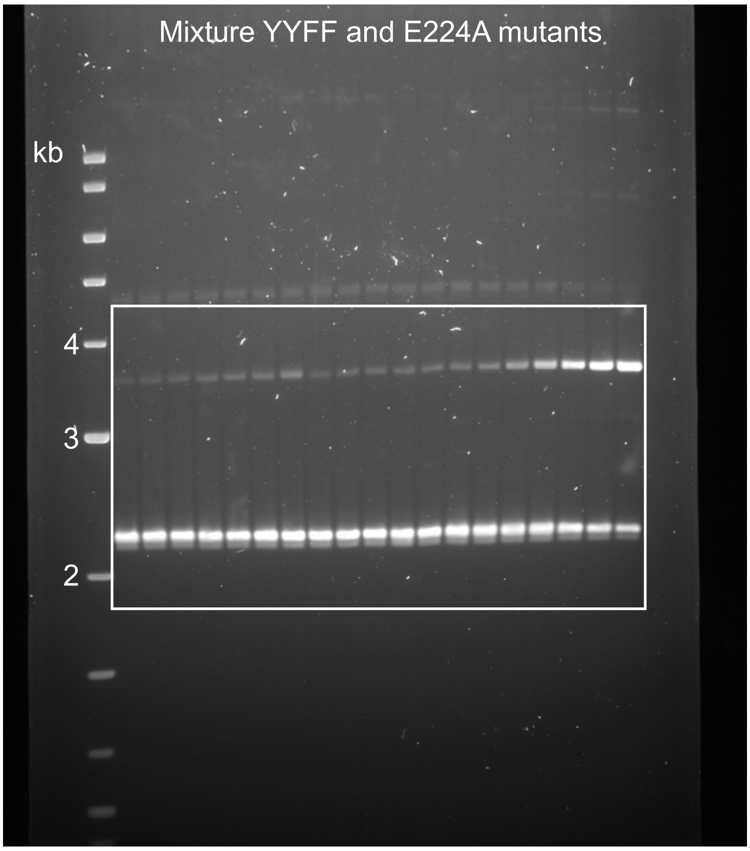

Used in Fig. 4b

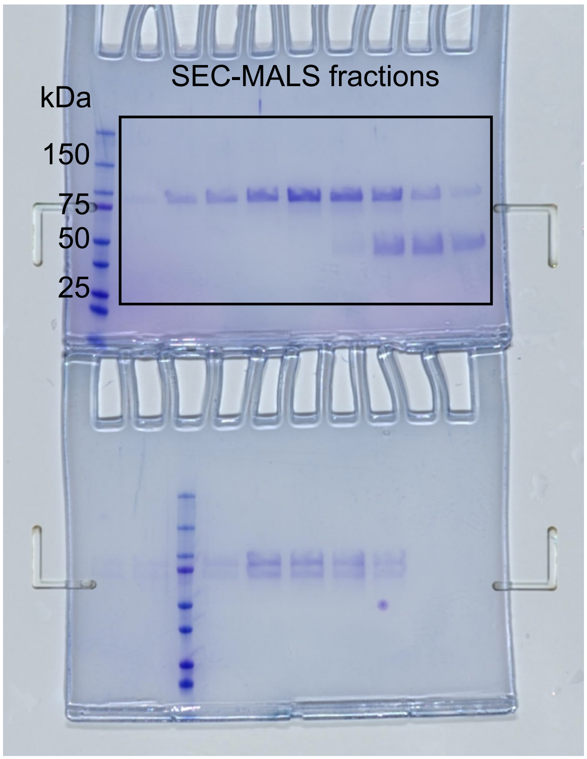

Used in Fig. 4c

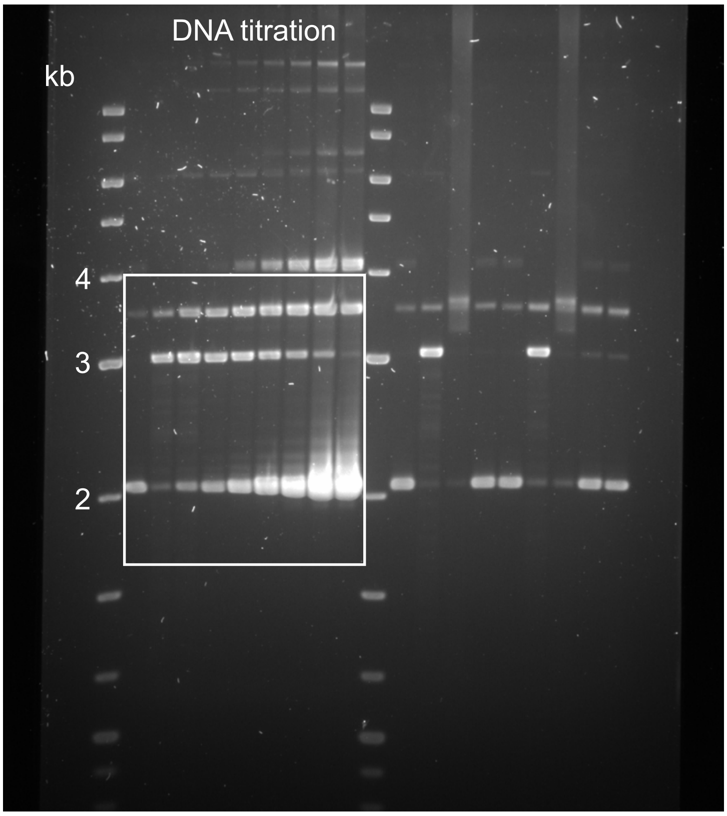

Used in Fig. 4d

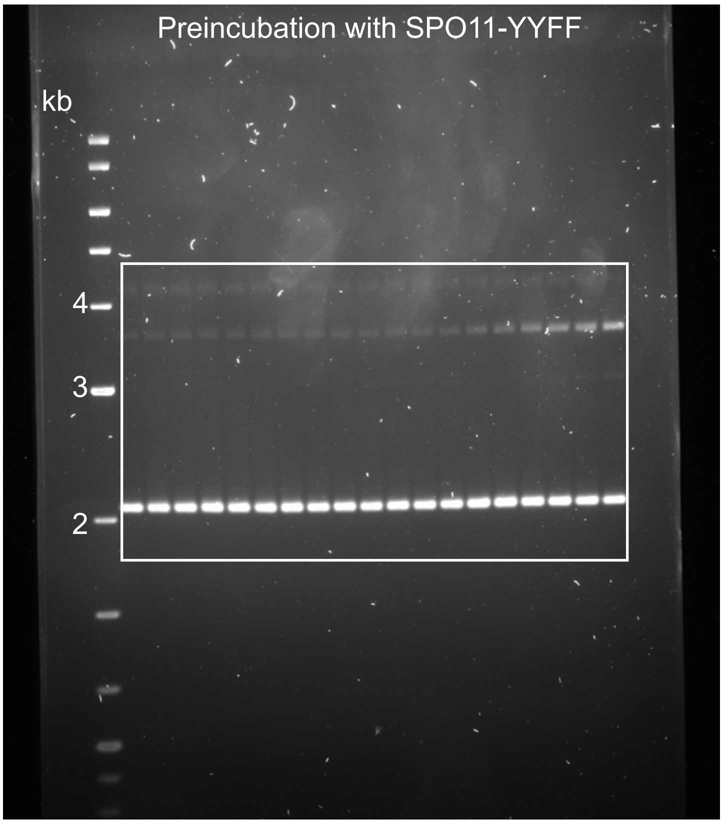

Used in Fig. 4e

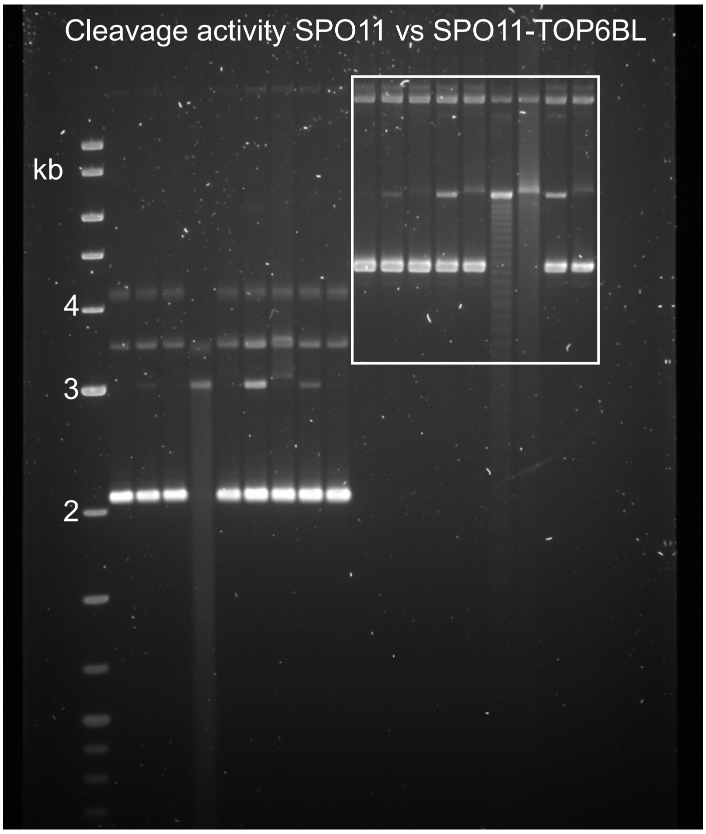

Used in Fig. 5d

DNA-binding activity SPO11 vs SPO11-TOP6BL

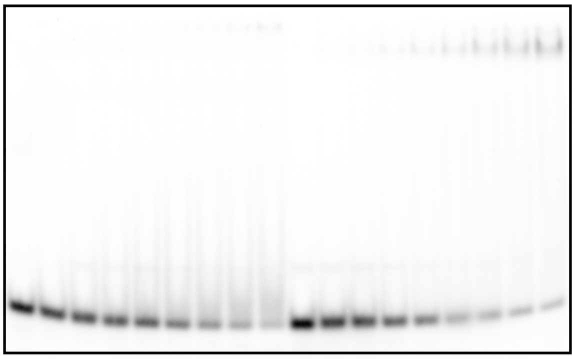

Used in Fig. 5e

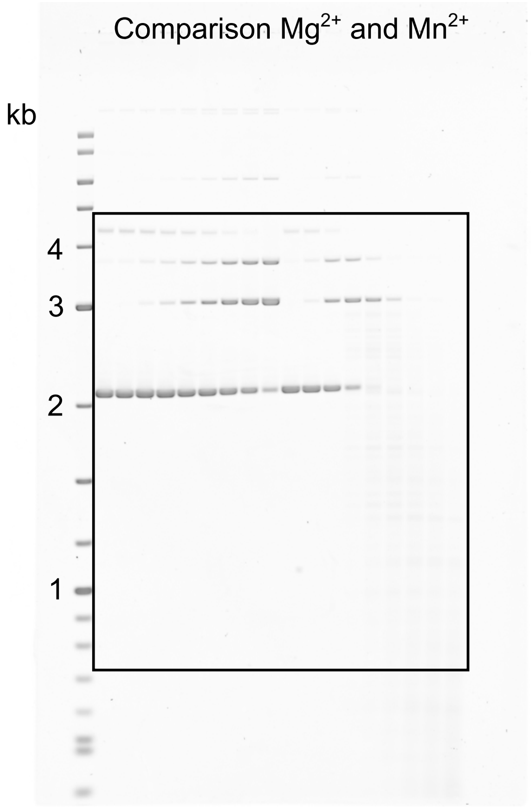

Used in Extended Data Fig. 1a

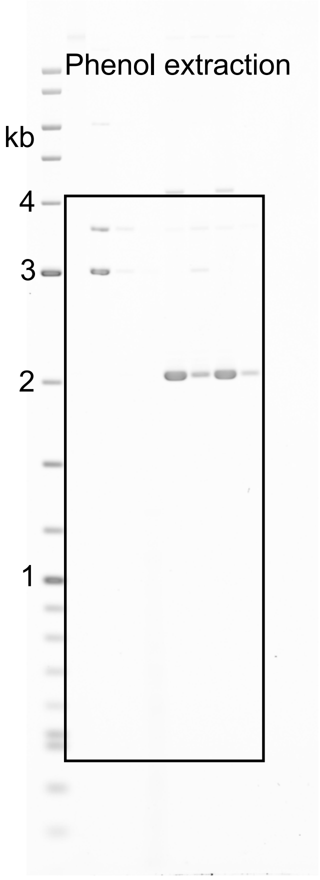

Used in Extended Data Fig. 1b

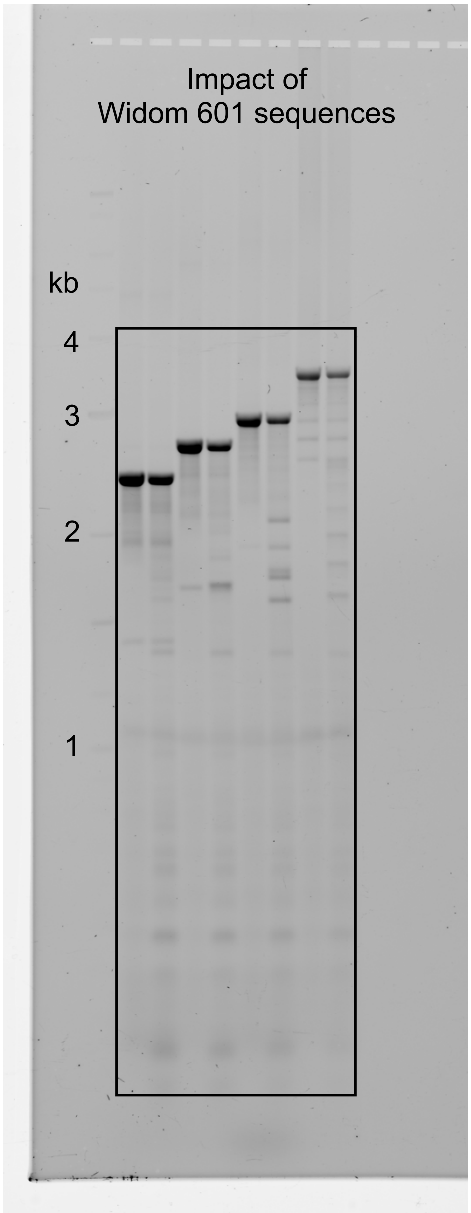

Used in Extended Data Fig. 2a

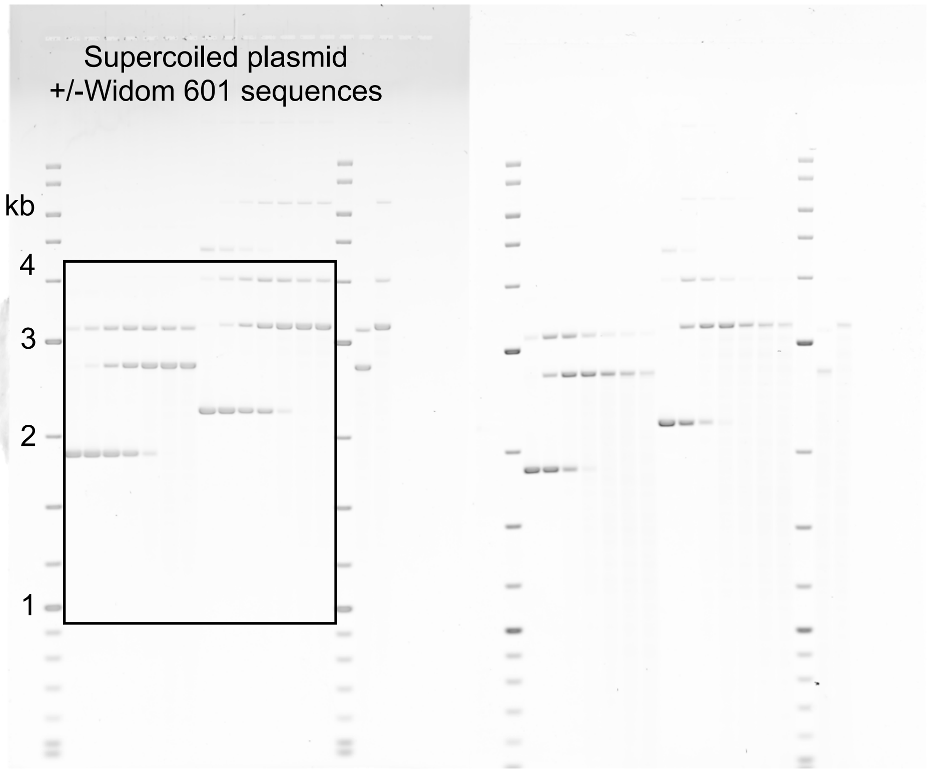

Used in Extended Data Fig. 3a

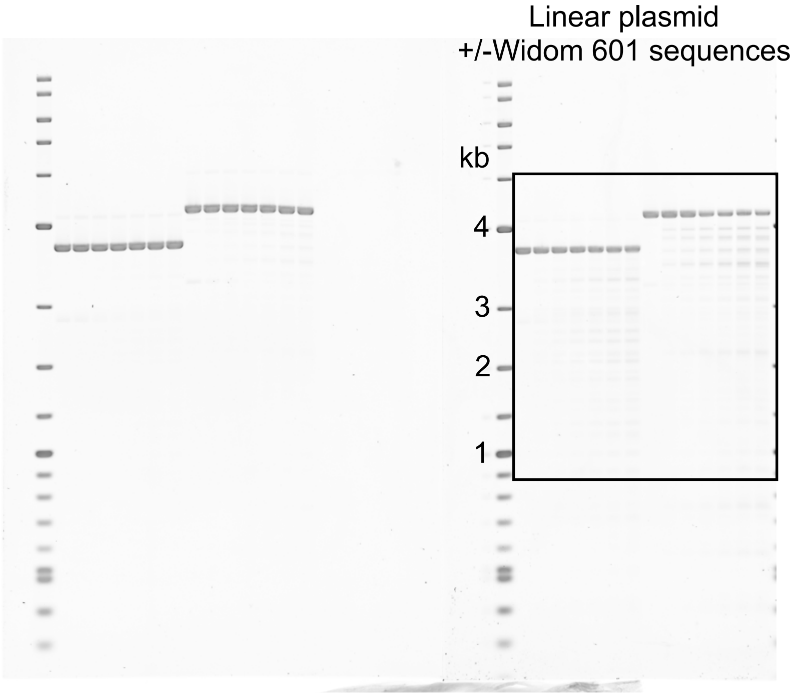

Used in Extended Data Fig. 3b

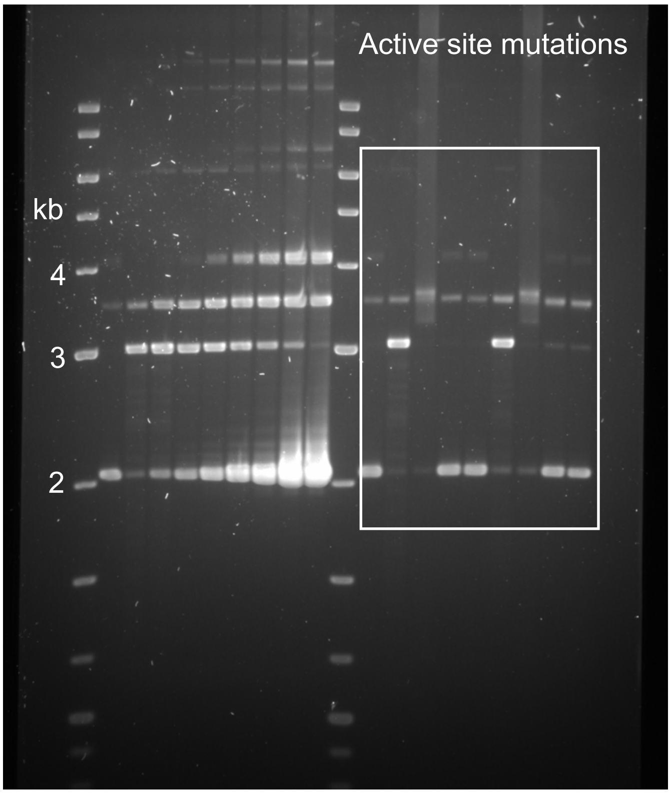

Used in Extended Data Fig. 4d

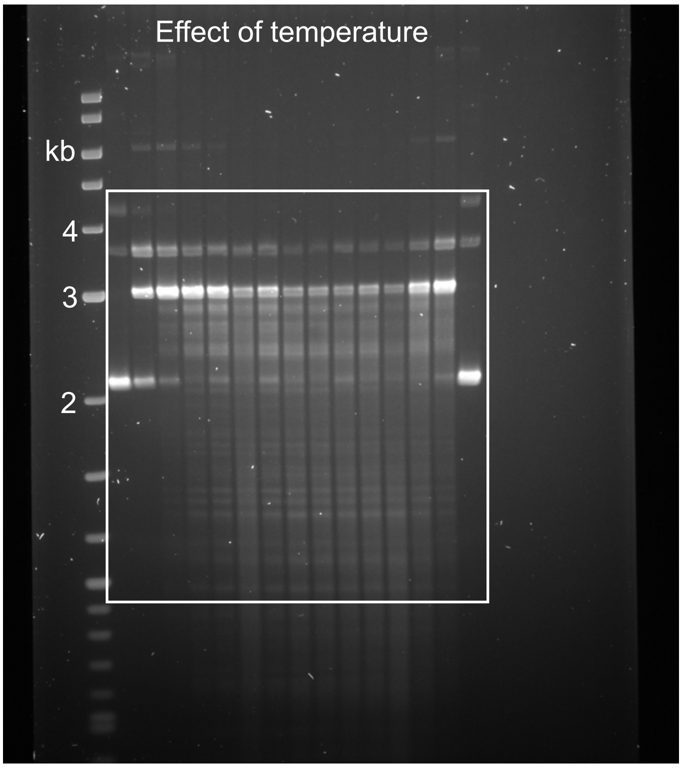

Used in Extended Data Fig. 5a

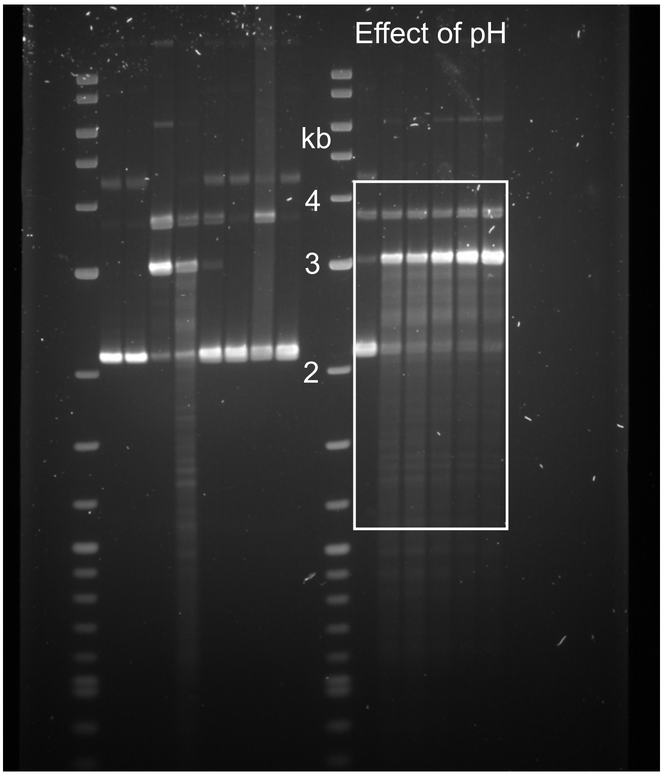

Used in Extended Data Fig. 5b

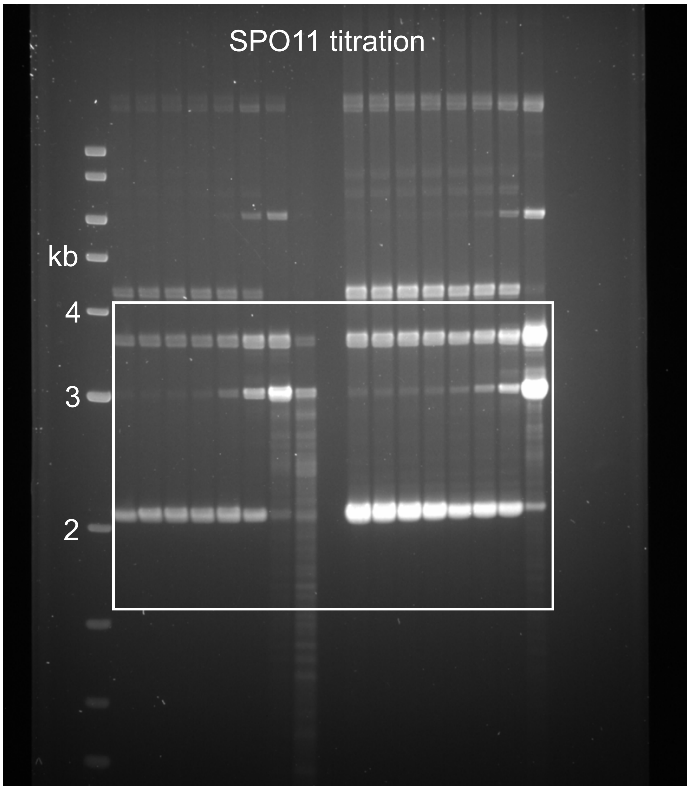

Used in Extended Data Fig. 5c

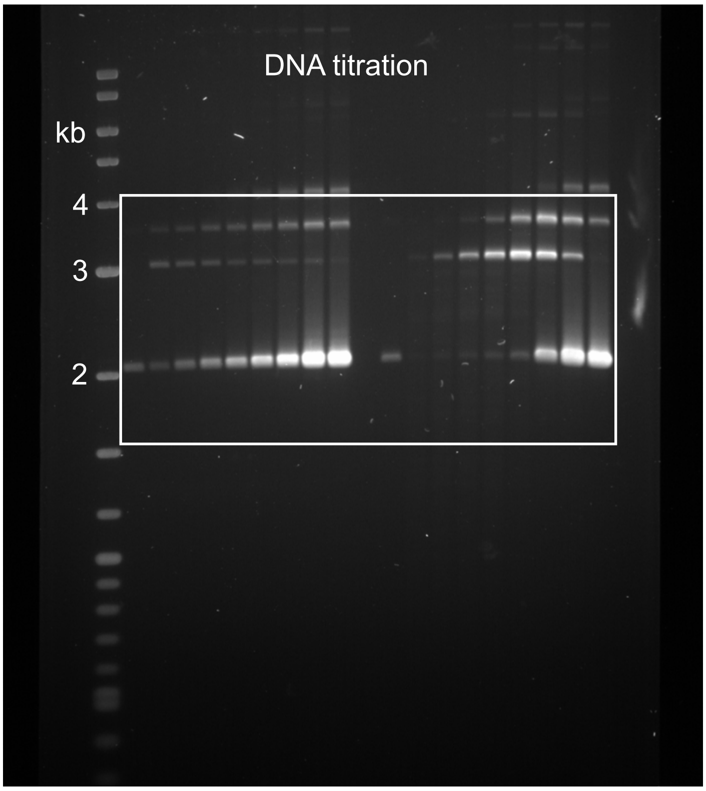

Used in Extended Data Fig. 5d

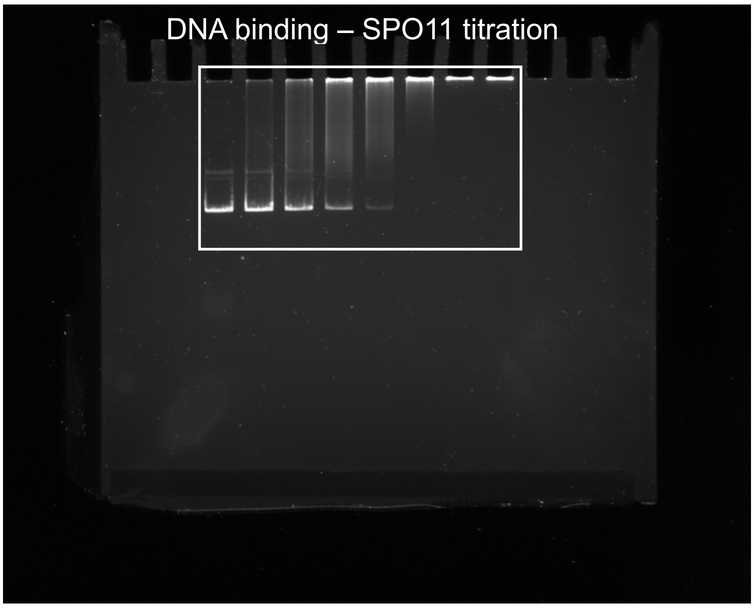

Used in Extended Data Fig. 6a

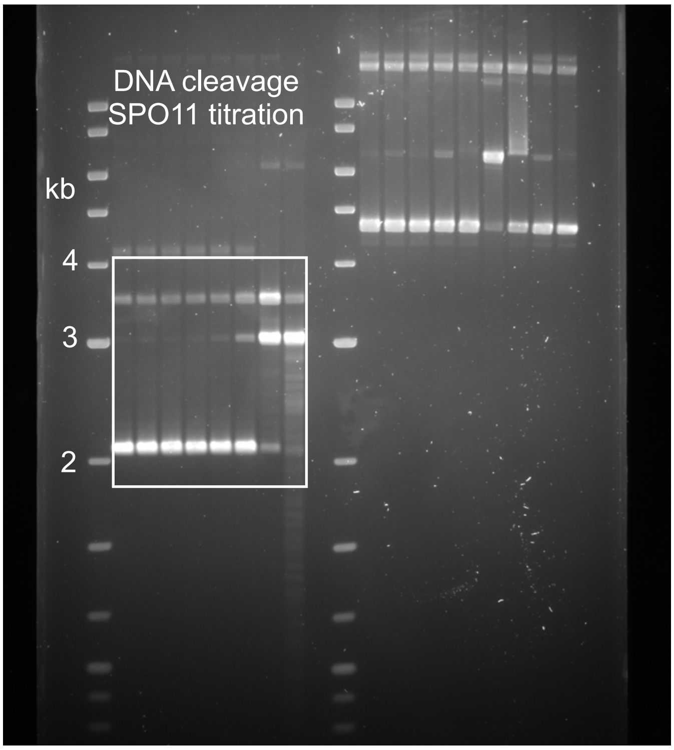

Used in Extended Data Fig. 6b

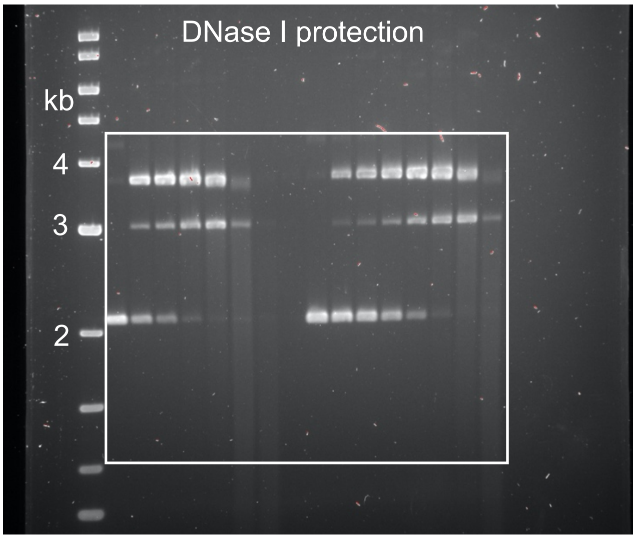

Used in Extended Data Fig. 6d

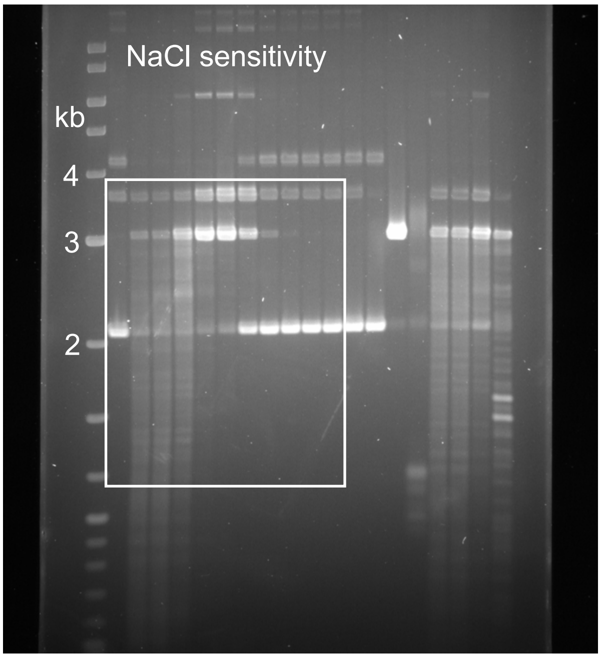

Used in Extended Data Fig. 6f

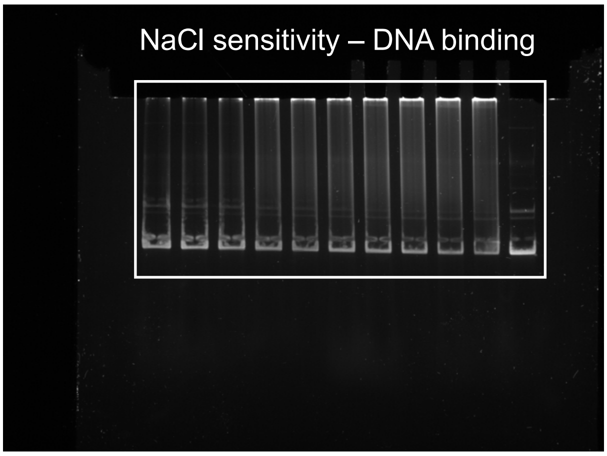

Used in Extended Data Fig. 6g

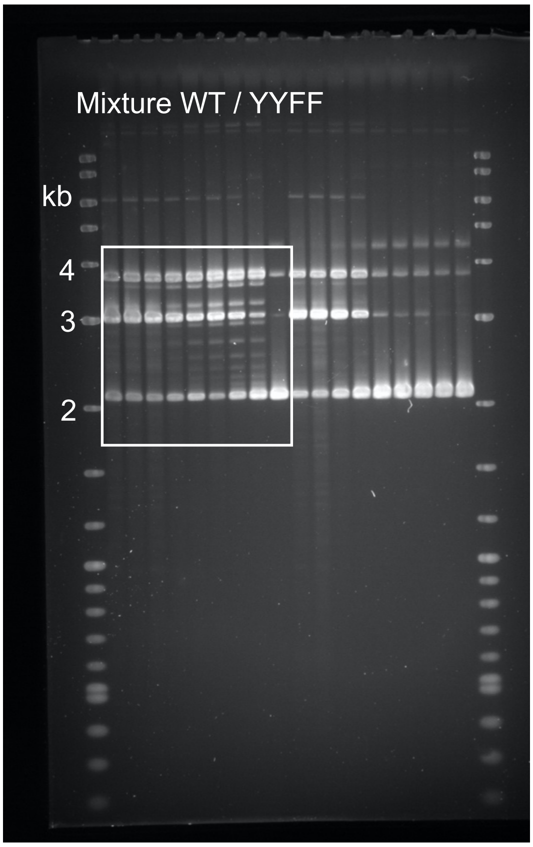

Used in Extended Data Fig. 7a

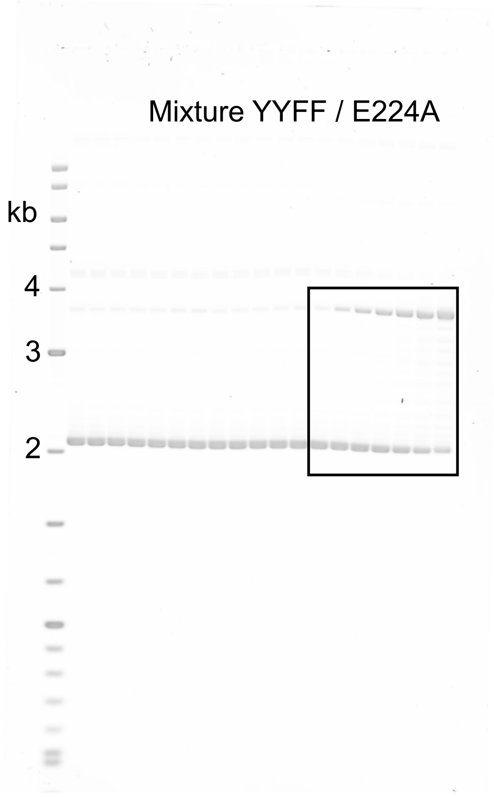

Used in Extended Data Fig. 7b

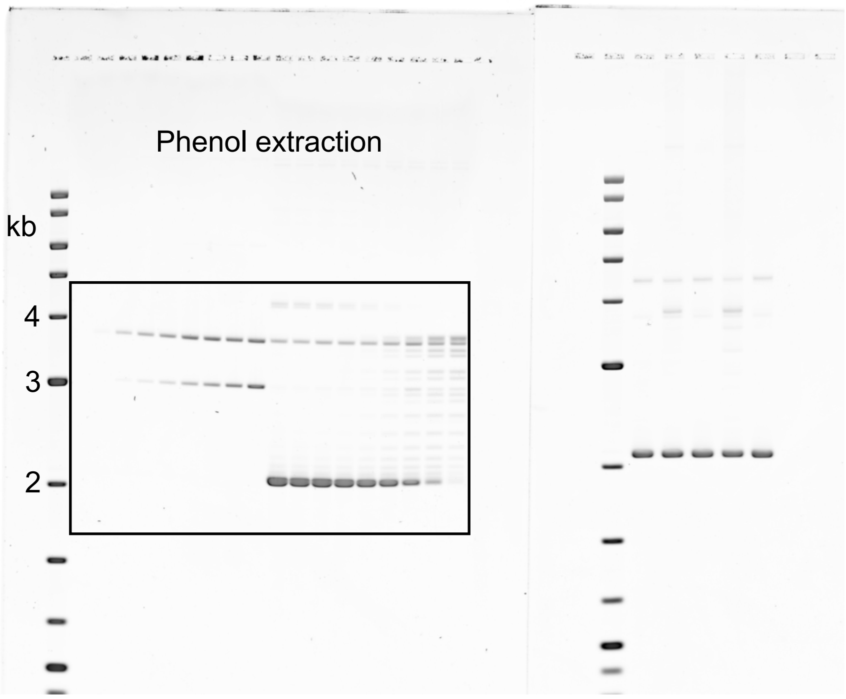

Used in Extended Data Fig. 7c
